# Use of glucarpidase (carboxypeptidase‐G2) in pediatric cancer patients: 11‐year experience of a tertiary center

**DOI:** 10.1002/jha2.799

**Published:** 2023-09-27

**Authors:** Rocío Vila, Alba Rubio‐San‐Simón, Pablo Zubiaur, Marcos Navares‐Gómez, Patricia Gómez‐Hernández, Begoña Arce, Luis Madero

**Affiliations:** ^1^ Oncohematology Unit Niño Jesús University Children's Hospital Madrid Spain; ^2^ Clinical Pharmacology Service La Princesa University Hospital Autonomous University of Madrid Madrid Spain; ^3^ Hospital Pharmacy Unit Niño Jesús University Children's Hospital Madrid Spain

**Keywords:** acute renal failure, carboxypeptidase, glucarpidase, methotrexate, pediatric oncology, pharmacogenetics

## Abstract

Methotrexate is an essential drug in the treatment of childhood cancer that is not exempt from toxicities. Glucarpidase is a drug used to reduce the toxic concentration of plasma methotrexate in patients with delayed elimination or at risk of toxicity. We describe the characteristics of a cohort of pediatric patients that received glucarpidase and analyze its role in the treatment of toxicity induced by high doses of methotrexate (HDMTX).

Retrospective observational study of all pediatric cancer patients who received glucarpidase between 2012 and 2022 at a single center.

Fifteen patients were treated with a single dose of glucarpidase, eleven of them presented with acute lymphoblastic leukemia and received HDMTX at 5 g/m^2^ in 24‐hour infusion. In eight patients, glucarpidase was administered during the first cycle of HDMTX. The indication in thirteen cases was acute renal failure with delayed elimination of plasma methotrexate. The median maximum creatinine was 1.22 mg/dl (0.68 2.01 mg/dl), with a median increase over its baseline level of 313%. All patients normalized renal function after glucarpidase administration, with a median methotrexate excretion time of 193 hours (42–312 hours). No grade ≥2 adverse events derived from carboxypeptidase administration. Eleven patients received new doses of HDMTX in subsequent cycles, without new episodes of serious toxicity.

The use of glucarpidase is effective and safe in the treatment of acute renal failure and methotrexate elimination delay in pediatric cancer patients. Further HDMTX doses may be prescribed without additional toxicities.

## Graphical abstract

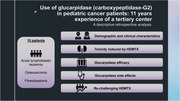

**Abbreviations**
ABCG2: ATP Binding Cassette Subfamily G Member 2
ALL: acute lymphoblastic leukemia
CA: California
CIR: clinically important reduction
CTCAE: Common Terminology Criteria for Adverse Events
DAMPA: 2, 4‐diamino‐N (10)‐methylpteroic acid
HDMTX: high doses of methotrexate
HPLC‐MS: liquid chromatography–mass spectrometry
HPLC‐MS/MS: liquid chromatography with tandem mass spectrometry
HPLC‐UV/VIS: high‐performance liquid chromatography‐ultraviolet
IM: intermediate metabolizers
ML: slow metabolizers
MN: normal metabolizers
MR: extensive metabolizers
MTHFR: methylenetetrahydrofolate reductase
NUDT15: Nudix hydrolase 15
PCR: polymerase chain reaction
PETHEMA: Programa Español de Tratamiento en Hematología
SEHOP: Sociedad Española de Hematología y Oncología Pediátricas
SLCO1B1: solute carrier organic anion transporter family member 1B1
UAM: Universidad Autonoma Madrid
UGT1A1: UDP‐glucuronosyltransferase 1‐1
USA: United States of America

## 1 INTRODUCTION

Methotrexate is a widely used chemotherapeutic agent in pediatric cancers such as acute lymphoblastic leukemia (ALL), osteosarcoma, and brain tumors. Methotrexate competitively inhibits the enzyme dihydrofolate reductase, decreasing the synthesis of tetrahydrofolate from folic acid, an essential cofactor in the production of methionine, thymidylate and purines, thus interfering with DNA synthesis [1, 2].

Treatment with high doses of methotrexate (HDMTX), greater than >500 mg/m^2^ in an infusion time between 2 and 36 h [3] is associated with a wide variety of toxicities. In some cases, these events are serious and may require reduction or event discontinuation of the methotrexate subsequent doses. Most frequent adverse events are myelosuppression, gastrointestinal, and mucocutaneous toxicities, which affect to most of the patients who receive treatment with methotrexate as an antineoplastic agent [4]. In addition, other toxicities such as acute renal toxicity may appear with an incidence that varies between 2% and 12% [4]. The association between mutations in genes that code for enzymes involved in the methotrexate's activity metabolic pathways and the toxicity induced by this drug is complex and not well stablished [5].

Several gene studies have described genetic variants that could be possibly associated with different toxicities induced by methotrexate in cancer patients [5, 6, 7, 8, 9]. Some of these associations have been identified with a moderate‐high level of evidence (PharmGKB levels 2a and 2B), as it is shown in Table 1 [10].

**TABLE 1:** Summary of polymorphisms in enzymes and transporters with moderate‐high level of evidence (2A‐2B) according to PharmaGKB.

Transporters			
Gene	SNP	Association	Level of evidence
*ABCB1*	rs1045642	Toxicity	2A
*SLCO1B1*	rs11045879	Toxicity	2A
*SLC19A1*	rs1051266	Toxicity	2A

Enzymes			
Gene	SNP	Association	Level of evidence
*MTHFR*	rs1801133	Toxicity, effectiveness, dose	2A
*ATIC*	rs4673993	Effectiveness	2B
*TYMS*	rs45445694	Effectiveness	2B

Among the measures aimed at preventing toxicity are the monitoring of plasmatic levels, the hyperhydration, the urinary alkalinization and the administration of calcium folinate [11, 12].

Although rare, methotrexate‐induced renal toxicity is a medical emergency, as it is associated with a delayed elimination of the drug, favoring greater tissue exposure to high concentrations and increasing the risk of toxicity [3, 13]. Glucarpidase (carboxypeptidase‐G2) is a recombinant bacterial enzyme that degrades circulating methotrexate into inactive metabolites (2,4‐diamino‐N(10)‐methylpteroic acid – DAMPA‐ and glutamate) with a better elimination profile and lower toxicity [3]. It is indicated to reduce the toxic concentration of plasma methotrexate in adults and children (28 days and older) with delayed methotrexate elimination or risk of toxicity [14, 15]. Immunological methods overestimate the levels of methotrexate after glucarpidase administration because they determine both the concentration of circulating methotrexate and its inactive metabolite DAMPA [16]. Measurement of methotrexate levels after glucarpidase administration should be performed using high‐performance liquid chromatography, such as the chromatograph coupled to a visible/ultraviolet detector (HPLC‐UV/VIS), coupled to mass spectrometry (HPLC‐MS), coupled to triple quadrupole mass spectrometry (HPLC‐MS/MS), among others [16].

We present our experience with the use of glucarpidase in a cohort of pediatric cancer patients receiving HDMTX and discuss potential risk factors associated with severe renal toxicity in these patients.

## 2 MATERIAL AND METHODS

A descriptive retrospective study was carried out by analyzing the medical records of all patients treated with HDMTX who received glucarpidase in our institution between January 2012 and December 2022. Epidemiological, clinical and analytics variables were collected. Toxic effects were graded according to the CTCAE version 5.0 criteria [17].

Methotrexate plasma concentration was determined by HPLC‐MS according to patient's protocol. It combines mass spectrometry with high‐performance liquid chromatography in order to identify drug metabolites without endogenous metabolites interference [18]. The time to complete methotrexate excretion was defined as the time between the start of the methotrexate infusion and the time when the plasma methotrexate concentration fell below 0.25 μM/L. Clinically important reduction (CIR) in plasma methotrexate was defined as methotrexate ≤1 μM/L at all postglucarpidase measurements [19].

Renal function recovery was assessed by serum creatinine values or creatinine clearance measurements after the HDMTX course.

Genotyping process implied DNA extraction from blood sample by an automated DNA extraction system (MagNa Pure System, Roche Applied Science), which was quantified with a Qubit 4 Fluorometer (ThermoFisher, USA). Automated analysis with the QuantStudio 12K Flex Real‐Time PCR System [20] (Applied Biosystems, Foster City, CA, USA) and OpenArray thermoblocks or 96‐fast were used for large genotyping.

The polymorphisms studied were some with higher evidence in PharmGKB [10] such as MTHFR and SLCO1B1 and other published such as ABCG2 [8], UGT1A1 [8], NUDT15 [9] as well as some cytochrome polymorphisms [21].

The analysis of the biological samples was carried out with the approval of the Hospital Niño Jesús ethics committee. Informed consent was collected from all participating patients, guaranteeing the confidentiality of the data, the right to access them and, in the event of having a significant implication for the patient's health and there being a possibility of improving it, providing genetic counseling according to what is included in article 47 of the Biomedical Research Law 14/2007 and in the Declaration of Helsinki.

Statistical analysis was performed using the SPSS v21 program. Quantitative variables were expressed as median and range and qualitative variables as percentage.

## 3 RESULTS

### 3.1 Population

Fifteen patients received glucarpidase, eight male and seven female, with a median age of 8.68 years (range 3.3–16.9 years).

The most frequent underlying malignancy was ALL in 11 patients. The distribution of the different cancer diseases of the population, additional characteristics of the patients and the anticancer treatment protocols are shown in Table 2.

**TABLE 2:** Patient's diagnosis and characteristics of their protocol in the cohort.

Diagnosis	Number of patients	Protocol	HDMTX dosage	Time HDMTX level measured	Leucovorin rescue indication
ALL	11	SEHOP‐PETHEMA 2013 [26]	5 g/m^2^/24H	24, 36, 42, 48 y 54H	Every 6H: since 42H until MTX level < < 0.25 μM/L
Localized Osteosarcoma	2	SEHOP‐SO‐2010 [35]	12 g/m^2^/4H	24, 48 y 68H	Every 6H: since 24 h until MTX level < 0.2 μM/L.
Mature B cell leukemia	1	Inter‐B‐NHL Ritux 2010 [36]	8 g/m^2^/24H	From 36H and every 6H until MTX < 0.15 μM/L.	Every 6H: since 36H until MTX levels < 0.15 μM/L.
Metastatic Pineoblastoma	1	HEAD‐START II [37]	400 mg/Kg/4H	From 24H and every 6H until levels < 0.1 μM/L.	Every 6H: since 24H until MTX levels < 0.1 μM/L.

Abbreviations: ALL, acute lymphoblastic leukemia; H, hours; HDMTX, high doses of methotrexate; MTX, methotrexate.

All patients received prophylactic measures to prevent methotrexate toxicity consisting in intravenous rehydration, urinary alkalinization, monitoring of methotrexate plasma levels, and administration of calcium folinate according to those levels.

### 3.2 Glucarpidase administration

Glucarpidase was administered during the first cycle of HDMTX in eight patients, in five patients during the second cycle, and in two patients during the fifth cycle of HDMTX. Four of seven patients who received glucarpidase beyond the first cycle had previously presented delayed elimination of methotrexate but without associated toxicity.

Thirteen patients received glucarpidase due to a grade 2–3 acute renal failure associated with delayed methotrexate elimination, one patient presented with grade 2–3 acute renal failure with adequate methotrexate clearance. The creatinine median peak during the episode in these patients was 1.22 mg/dL (range 0.68 ‐ 2.01 mg/dL), with a median increase over baseline of 313% (range 186%–650%). All of them normalized renal function once the episode resolved. One patient received glucarpidase due to grade 3 neurotoxicity. He developed irritability and fluctuating level of consciousness, with adequate renal function and methotrexate plasma levels within the normal range according to the treatment protocol. Neurotoxicity was completely resolved.

In all cases, carboxypeptidase was administered within the first 48 h from the start of the methotrexate infusion: in six cases in the first 24 h after methotrexate infusion, in one case 30 h, in seven cases 36 h and in one case 48 h after infusion. The median time elapsed until methotrexate plasma levels were <0.25 μM/L was 193 h (range 42–312 h). Glucarpidase was administered in a single intravenous dose of 50 units/kg, with a median patient's weight of 33 Kg (14.8–88 Kg). Only one patient presented a side effect derived from carboxypeptidase administration, pruritic rash grade 1.

The median 24h‐MTX level was 101.4 μM/L (38.2–296.96 μM/L), the median 36h‐MTX level was 6.07 μM/L (0.11–62.8 μM/L). The median 42h‐MTX level was 2.76 μM/L (0.09–6.92 μM/L). The median 48h‐MTX level was 1.22 μM/L (0.08–4.66 μM/L) and the median 72h‐MTX level was 0.8 μM/L (0.03–2.28 μM/L).

Six patients reached CIR, although one of them did not have delayed methotrexate clearance (five patients, 33%). The median preglucarpidase MTX level was 63.2 μM/L (1.74‐296.9 μM/L) and the median postglucarpidase MTX level was 3.44 μM/L (0.2–15.4 μM/L), with a median methotrexate reduction of 92.6% (77.5%−99.42%).

### 3.3 Methotrexate dose modification

**FIGURE 1:**
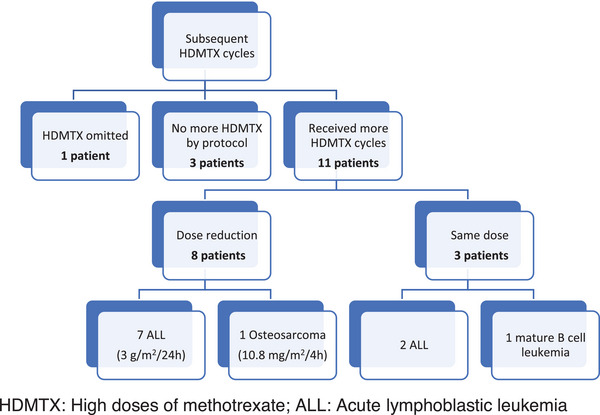
Chart of the subsequent high doses of methotrexate cycles. ALL, acute lymphoblastic leukemia; HDMTX, high doses of methotrexate.

Subsequent treatment with HDMTX was maintained in 11 patients, in nine of them at a reduced dose. Three patients did not receive new cycles of methotrexate according to the treatment protocol and in one patient the remaining cycles of methotrexate were omitted. Figure 1 shows the characteristics of methotrexate dose modifications.

None of them presented new methotrexate‐related serious toxicities in subsequent administrations of HDMTX.

### 3.4 Polymorphism analysis

Study of the enzyme methylenetetrahydrofolate reductase (MTHFR) polymorphisms was carried out in four patients. Two patients were homozygous for the MTHFR AA1298C variant (both had acute renal toxicity and delayed methotrexate elimination); one patient heterozygous for the MTHFR C677T variant (he presented with renal toxicity and delayed methotrexate elimination) and another patient was negative for both variants.

In five patients were analyzed the phenotypes of the following genes involved in the methotrexate's activity metabolic pathways: Solute carrier organic anion transporter family member 1B1 (SLCO1B1) gene with four normal metabolizers (MN) and one intermediate (IM); ATP Binding Cassette Subfamily G Member 2 (ABCG2) gene with four normal and one IM; UDP‐glucuronosyltransferase 1‐1 (UGT1A1) gene with one normal and two IM; and Nudix hydrolase 15 (NUDT15) gene, that was normal in all cases. The analysis of the genes that code for cytochrome P450 enzymes was in the case of CYP2B6, one MN, three IM, and two extensive metabolizers (MR) were identified; CYP2D6 three MN, one IM and one slow (ML), CYP3A5 two IM and three ML, CYP2C19 two MN, one IM and two MR, all being MN in the case of CYP2C9.

## 4 DISCUSSION

Toxicities associated with HDMTX treatment are varied and potentially serious. In the case of severe acute renal failure, it constitutes a medical emergency, since it can increase the concentrations of methotrexate in the blood and cause additional toxicities. In this study we present a detailed description of all pediatric cancer patients treated with glucarpidase due to side effects derived from HDMTX treatment in our center. Glucarpidase provided a safe and effective therapeutic option in these patients, achieving complete resolution of the toxicity in all cases.

Traditionally, the HDMTX administration protocol in osteosarcoma (12 g/m^2^ in 4‐h infusion) is the one that has been associated with a higher incidence of renal failure and delayed methotrexate elimination [22]. However, in our cohort, only two patients with osteosarcoma required glucarpidase, the most frequent indication was ALL receiving a regimen of 5 g/m^2^ in 24‐h infusion, as occurs in other series [23, 24]. The fact that ALL is the most frequent neoplasm in the pediatric population [25] and that four cycles of HDMTX (5 g/m^2^) are administered as consolidation treatment according to the current SEHOP‐PETHEMA national treatment protocol [26] probably influenced on the result. No patient with lymphoma required glucarpidase in our center, despite being the third most common group of tumors in the pediatric population [25] and being treated with HDMTX [27, 28].

As in the Svahn et al. cohort [24], almost half of the patients required glucarpidase in the first cycle of HDMTX, although unlike other groups, no patient required more than one dose of glucarpidase during the toxicity episode or in subsequent HDMTX cycles. As recommended by user guides [3, 26], glucarpidase infusion was administrated in the first 48–60 h from the start of treatment with methotrexate. It has been described that delays beyond 96‐h increase the risk of grade 4 and 5 toxicity [11].

In relation to renal toxicity, no patient presented oligoanuria or required extracorporeal purification techniques, unlike other case series and publications [23, 24, 29]. The creatinine peak during the toxicity episode was lower than in other reported experiences [11, 23, 24]. As described in the literature, all patients recovered normal renal function after the toxicity episode, with no mortality associated with methotrexate toxicity [3, 23, 24].

Regarding the neurotoxicity produced by methotrexate, the efficacy of glucarpidase administration is limited given its difficulty in crossing the blood‐brain barrier [30], Despite this, a patient at our center received glucarpidase for an episode of neurotoxicity without renal failure or delayed elimination of methotrexate, with complete resolution of the neurotoxicity. Glucarpidase was administered according to the medical criteria of his referring physician. However, it is difficult to assess its effect in this patient as the neurotoxicity could have disappeared without its administration.

It is important to remember that, after the administration of carboxypeptidase, the determination of methotrexate levels by immunological methods overestimates its value. In the presented cohort, the median time to reach plasma levels less than 0.25 μM/L as measured by LC‐MS/MS was 193 h. Other groups have reported similar methotrexate clearance times after glucarpidase administration, although comparisons are limited by the cut‐off points for methotrexate plasma levels and the laboratory techniques used to determine it [23, 24].

In order to determine its efficacy, similar to published in the literature for this age range, five patients (33%) reached CIR [1]. We found a 92.59% reduction of methotrexate levels after first dose of glucarpidase, slightly lower than it is described. 19, 24]. Hence the importance of properly identifying patients who can benefit from this treatment, as well as assessing the need for second doses in selected patients.

Dose reduction of methotrexate after severe renal toxicity requiring glucarpidase is controversial. The literature recommends safely resuming treatment with HDMTX at full doses, both in pediatric and adult patients, after recovery of normal renal function in patients who have received glucarpidase [3, 23, 24]. It has also been described that patients who have required glucarpidase once, usually present better renal function and lower creatinine levels in subsequent cycles [23] probably in relation to more aggressive prophylactic measures.

In this respect, the analysis of the polymorphisms of the genes involved in the metabolism and mechanism of action of methotrexate is of particular importance.

Several gene studies have found genetic variants that could be possibly associated with different toxicities induced by methotrexate in cancer patients [5, 6, 7]. Some of these associations have been identified with a moderate‐high level of evidence (PharmGKB levels 2a and 2B) [10].

These associations are established between polymorphisms located in genes that encode for enzymes related to methotrexate's mechanism of action or in genes that encode for transporters, which determine its pharmacokinetics. However, the association between germline variations and methotrexate‐induced toxicities is complex and the evidence is contradictory, so the implementation of pharmacogenetics in methotrexate's clinical practice is limited. There are not adjustment guidelines, dose or change of treatment. For this reason, in the two patients in our cohort with MTHFR rs1801133 (C > T) T/T genotype, and in the patient with the MTHFR rs1801131 (T > G) T/G genotype, the subsequent clinical management was not modified.

In our study alleles were defined according to PharmVar core allele definitions, available at *pharmvar.org*. Genotype‐informed phenotyping was carried out following CPIC guidelines [31] for the different genes which are consistent with PharmGKB Gene‐Specific tables [32] in order to define slow, intermediate and extensive metabolizers for each gene.

Regarding cytochrome polymorphisms, the European and American drug labels of methotrexate do not provide much information on the pharmacology of the drug [21]. Some claims suggest CYP‐mediated metabolism, but the paucity of data to support (or refute) them is clear. Methotrexate is partially metabolized by intestinal flora after oral administration, so it could be hypothesized intestinal CYPS could play a role. It primarily undergoes hepatic and intracellular metabolism to active polyglutamated forms which can be converted back to methotrexate by hydrolase enzymes and it also undergoes minor metabolism to active 7 hydroxymethotrexate [21]. The fact that MTX is hydroxylated to 7‐hydroxymethotrexate suggests CYP‐mediated metabolism.

Every studied gene has already been related to methotrexate [7, 8, 9] so our descriptive study propose analyzing the genotype‐informed pharmacogenetic phenotype is a good alternative to in vitro assays to establish new gene‐enzyme interactions, particularly for old drugs like methotrexate, as previous studies of this group suggest [33, 34].

As limitations, it should be highlighted those associated with the retrospective nature of the study, without being able to extract causal relationships from it. For this reason, additional studies are needed to correlate the role of these polymorphisms in methotrexate toxicity and allow dose adjustment in pediatric patients.

No predictors of delayed clearance of HDMTX were analyzed in pediatric patients except for the presence of a history of delayed clearance or prior toxicity from HDMTX. The presence of mucositis and myelosuppression was not assessed. Anticancer treatment protocols in general combine drugs with overlapping medullar and mucous toxicity that difficult the association of these toxicities with HDMTX [23, 24].

As conclusion, glucarpidase is an effective drug in pediatric patients with severe renal toxicity and delayed methotrexate elimination, lowering methotrexate levels and favoring the recovery of renal function. In addition, glucarpidase administration is safe in pediatric population, with infrequent and mild adverse effects.

## AUTHOR CONTRIBUTIONS

All authors have participated in the design, data collection, and writing of the manuscript.

## CONFLICT OF INTEREST STATEMENT

The authors declare that there is no conflict of interest that could be perceived as prejudicing the impartiality of the research reported.

## ETHICS APPROVAL STATEMENT

This study was carried out with the approval of the Hospital Niño Jesús ethics committee.

## PATIENT CONSENT STATEMENT

Informed consent was collected from all participating patients, guaranteeing the confidentiality of the data.

## CLINICAL TRIAL REGISTRATION

The authors have confirmed clinical trial registration is not needed for this submission.

## Data Availability

The data that support the findings in this study are available on request to the corresponding author (RV).
